# Socioeconomic Factors and Survival of Multiple Myeloma Patients

**DOI:** 10.3390/cancers13040590

**Published:** 2021-02-03

**Authors:** Kamal Chamoun, Amin Firoozmand, Paolo Caimi, Pingfu Fu, Shufen Cao, Folashade Otegbeye, Leland Metheny, Seema Patel, Stanton L. Gerson, Kirsten Boughan, Marcos De Lima, Ehsan Malek

**Affiliations:** 1 Hematologic Malignancies and Stem Cell Transplant Program, University Hospitals Seidman Cancer Center, Case Western Reserve University, Cleveland, OH 44106, USA; K_chamoun@outlook.com (K.C.); amin.firoozmand@uhhospitals.org (A.F.); paolo.caimi@case.edu (P.C.); folashade.otegbeye@UHhospitals.org (F.O.); Leland.Metheny@uhhospitals.org (L.M.); seema.patel@uhhospitals.org (S.P.); slg5@case.edu (S.L.G.); kirsten.boughan@uhhospitals.org (K.B.); marcos.delima@UHhospitals.org (M.D.L.); 2 Department of Population and Quantitative Health Sciences, Case Western Reserve University, Cleveland, OH 44106, USA; pxf16@case.edu (P.F.); shufen.cao@case.edu (S.C.)

**Keywords:** multiple myeloma (MM), autologous stem cell transplant (ASCT), overall survival (OS)

## Abstract

**Simple Summary:**

Multiple Myeloma is the third most common hematologic malignancy. Outcomes have improved significantly, as the result of the introduction of novel agents as well as higher utilization of autologous Hematopoietic Cell Transplant. These complex treatment regimens are quite expensive and their full applicability in the real world remains largely unknown. Several studies have shown that survival in patients with other types of cancer is influenced by their insurance, demographic factors, as well as socioeconomic status. Here we intend to examine the interplay of insurance status and other potential socioeconomic factors driving survival disparity for Multiple Myeloma patients. We used the National Cancer Database (NCDB) to assess the potential influence of these factors in this population.

**Abstract:**

Background: Outcome of Multiple Myeloma (MM) patients has improved as the result of the introduction of novel medications and use of autologous hematopoietic cell transplantation. However, this improvement comes at the expense of increased financial burden. It is largely unknown if socioeconomic factors influence MM survival. Methods: We used the National Cancer Database, a database that houses data on 70% of cancer patients in the US, to evaluate the effect of socioeconomic factors on the survival of 117,926 MM patients diagnosed between 2005 and 2014. Results: Patients aged ≥65 years who were privately insured lived longer than patients with Medicare (42 months vs. 31 months, respectively, *p <* 0.0001). Treatment in academic institutions led to better survival (HR: 1.49, 95% CI: 1.39, 1.59). Younger age, fewer comorbidities, treatment in academic centers, and living in a higher median income area were significantly associated with improved survival. After adjusting for confounders, survival of Medicare patients was similar to those with private insurance. However, the hazard of death remained higher for patients with Medicaid (HR: 1.59, 95% CI: 1.36, 1.87) or without insurance (HR: 1.62, 95% CI: 1.32, 1.99), compared to privately insured patients. Conclusion: Economic factors and treatment facility type play an important role in the survival of MM patients.

## 1. Introduction

Multiple Myeloma (MM) is the third most common hematologic malignancy with an estimated 32,110 new cases in 2019 (1.8% of all new cancer cases in the USA) [1]. The five-year overall survival (OS) improved from 24.6% to 52.4% for patients diagnosed from 1975 to 2014 [2]. This remarkable improvement was the result of the introduction of novel medications [3,4,5,6] that replaced traditional chemotherapy, in addition to the increased use of autologous Hematopoietic Stem Cell Transplantation (HSCT) [7,8,9]. These treatments are complex and expensive, and patients rely primarily on health insurance to cover the costs [10]. However, the patient’s share of the costs differs widely according to the insurance type. Several studies have shown that survival in patients with other types of cancer is influenced by their insurance status [11,12,13,14,15,16,17]. Considering that MM patients are the largest cancer subgroup treated with oral antineoplastic agents [18] and given the costs of these medications [18,19], there is an unmet need to examine the interplay of insurance status and other potential socioeconomic factors driving outcome disparity. Specifically, we hypothesized that insurance type may affect OS of MM patients in the USA independent of other factors. We used the National Cancer Database (NCDB) to test this hypothesis and reviewed the effects of potential financial toxicity in this population.

## 2. Results

The study included 117,926 patients. Patients’ characteristics are presented in Table 1. Fifty-three percent had Medicare, 35% had private insurance, 5.4% had Medicaid, 1% had other government insurance, while 3.2% were uninsured at time of diagnosis. Median age was 67 years. As expected, Medicaid and uninsured groups had significantly higher proportions of patients living in areas where the median income was less than 46k per year, compared to Medicare and private insurance groups. Among patients with Medicare, 33% were treated in an academic institution vs. 48% and 50% of patients with private insurance and Medicaid, respectively (*p <* 0.001). Nine percent of patients with Medicare were treated in large facilities (reported >50 cases/year) compared to 20% of patients with private insurance. Ninety-six percent of patients were treated in facilities located ≤120 miles from their area of residence (estimated as ≤2 h driving distance). More patients with private insurance traveled >120 miles to the treatment facility than patients with Medicare (5.7% vs. 3%, *p <* 0.0001). Among patients younger than 65 years, 33% of patients with private insurance received HSCT, compared to 20% of those on Medicare (*p <* 0.0001). For those 65 years and older, 11% of privately insured patients underwent HSCT, compared to 6% of those on Medicare (*p <* 0.0001).

**Figure 1:**
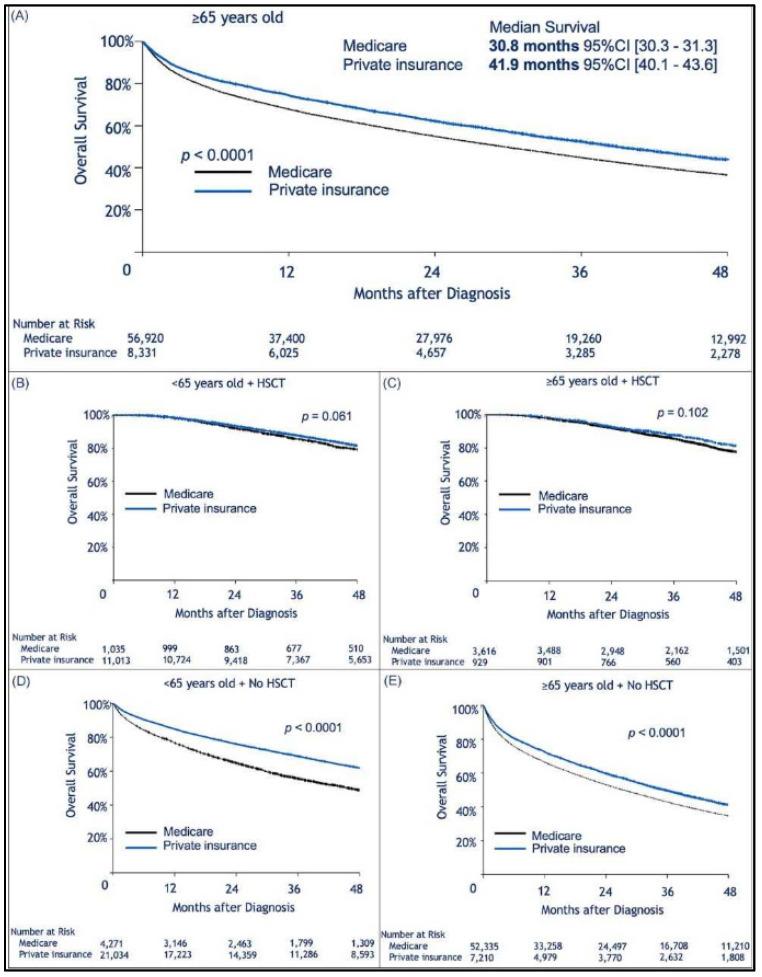
Kaplan–Meier estimation of overall survival comparing patients insured by Medicare or with private insurance. In (**A**) Among patients aged ≥65 years, private insurance was associated with longer survival; (**B**,**C**) No insurance-related difference in survival was observed for patients who received an autologous transplant. In (**B**), patients younger than 65 years old who received Hematopoietic Stem Cell Transplantation (HSCT), and in (**C**), patients ≥65 years old who received HSCT; however, for patients who did not receive a transplant, the influence of insurance type was again present. In (**D**), patients younger than 65 years old who did not undergo autologous transplant, and in (**E**) patients ≥65 years old who did not receive autologous transplant.

**Figure 2:**
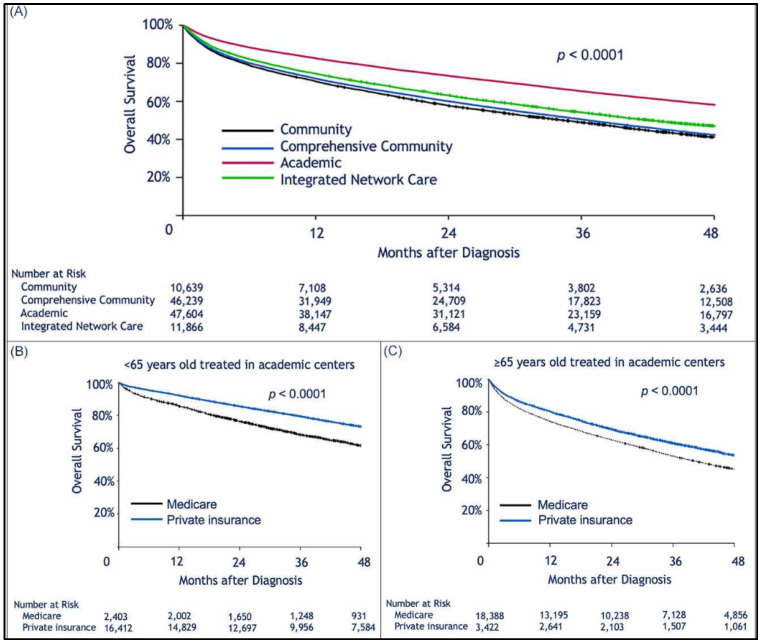
Kaplan–Meier estimation of overall survival comparing patients treated in different types of treatment centers. Treatment in an academic facility was associated with longer survival of multiple myeloma patients. However, privately insured patients treated at such facilities had better survival than those insured by Medicare. Kaplan–Meier estimation of overall survival as a function of: (**A**), treatment facility type; (**B**,**C**), show survival of patients privately insured or insured by Medicare who were treated at academic facilities; (**B**), patients aged <65 years; (**C**), patients ≥65 years old.

**Figure 3:**
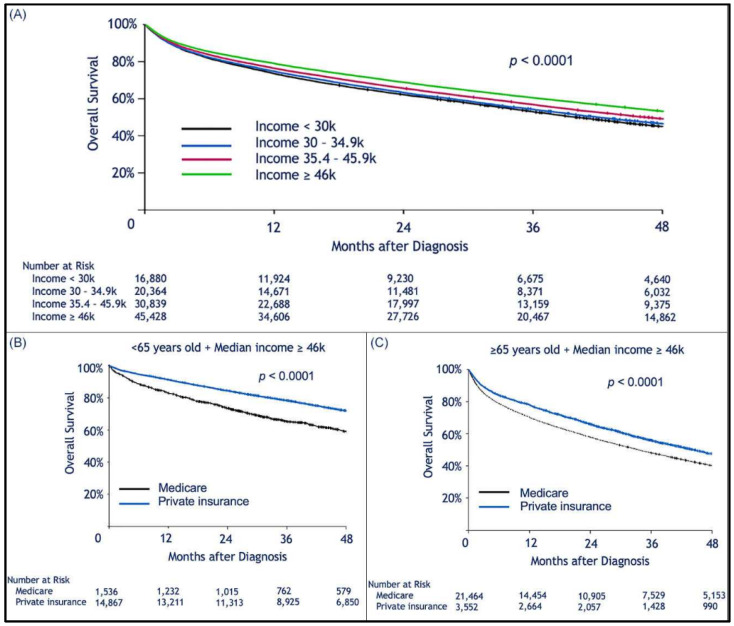
Kaplan–Meier estimation of overall survival comparing patients living in areas with different median household incomes. Higher median income was associated with longer survival after a diagnosis of multiple myeloma. Kaplan–Meier estimation of overall survival as a function of median (zip-code-based) income (**A**); among patients in the higher median income bracket, patients insured by Medicare had worse survival than those with private insurance (**B**,**C**); this effect was seen in the subgroup of patients <65 years old (**B**), and in the cohort of patients ≥65 years old.

Median follow-up was 30.2 (range, 0–145.2) months. When limiting the comparison to patients who were 65 years or older, we found a statistically significant survival advantage for patients with private insurance vs. those with Medicare (Figure 1A). Median OS for privately insured patients was 41.9 months (95% CI: 40.1–43.6) compared to 30.8 months (95% CI: 30.3–31.3) for patients with Medicare (*p <* 0.0001). When considering patients who received HSCT, there was no difference in survival between privately insured and Medicare patients regardless of age (Figure 1B,C). However, for those who did not receive HSCT there was a survival advantage for those with private insurance over Medicare (Figure 1D,E). We assume that autologous transplant recipients are less likely to rely on continuous or intermittent intensive medical therapy than patients who did not receive HSCT (Figure 1D,E). As previously shown, patients treated in academic institutions were found to survive significantly longer than those treated in community-based institutions (Figure 2A). Among patients treated in academic institutions, those with private insurance had longer survival than those with Medicare. This was observed in patients younger than 65 and those who were 65 or older (Figure 2B,C). Patients living in areas with higher median income had longer OS (Figure 3A). In addition, among those living in the higher median income areas, private insurance was associated with longer OS, regardless of age (Figure 3B,C).

There were racial differences in the insurance coverage type. For patients self-identified as black, there was a lower proportion of Medicare (47% vs. 55%; *p <* 0.0001), and private insurance (33% vs. 35%; *p* = 0.0008) and a higher percentage of Medicaid (10% vs. 4%; *p <* 0.0001) or uninsured patients (6% vs. 2%; *p <* 0.0001) compared to patients self-identified as white. A larger proportion of blacks had a Charlson score ≥1 (28.7 vs. 22.9 in whites; *p* < 0.0001) and lived in low-income regions (76.1% vs. 56.4% of whites; *p <* 0.0001). However, unadjusted OS was longer for African Americans compared to whites (50.6 months (95% CI: 49.1–52.3) vs. 46 months (95% CI: 45.4–46.6), respectively, (*p <* 0.0001)) as shown in Figure S1. Interestingly, a higher proportion of blacks were treated at academic medical centers (46.7% vs. 38.7%; *p <* 0.0001) and lived closer to treatment facilities (80.8% within 20 miles vs. 67.3% of whites; *p <* 0.0001). After adjusting for all above variables, black and white patients had similar OS. Furthermore, survival improved throughout the study period. When dividing the patients into three groups according to year of diagnosis we found that median survival was 35.1, 46.1, and 52.9 months for white patients diagnosed in 2005–07, 2008–10, and 2011–14, respectively. Likewise, median survival was 36.5, 51.2, and 60.3 months for black patients diagnosed in the same years.

We then performed a multivariable analysis that included age, gender, race, disease type (primary vs. secondary), Charlson score, type of treatment facility, median (zip-code-based) income, and type of insurance (Table 2 for all cohort and Table 3 for patients ≥65 years old). Disease stage was not included due to missing data in a large number of patients (89%). As expected, older age and higher number of comorbidities were associated with worse survival. Interestingly, higher (zip-code-based) median income and receiving treatment in academic hospitals were highly predictive of better survival. After controlling for the effects of these confounders, the survival difference between privately insured patients and Medicare recipients lost its statistical significance. However, this difference remained highly significant for patients with Medicaid or those without insurance at diagnosis. Compared to patients with private insurance, the hazard of death was increased by 59% for patients with Medicaid (*p <* 0.0001) and by 62% for those without insurance at diagnosis (*p <* 0.0001).

## 3. Discussion

MM is the leading cancer type being treated with oral antineoplastic [18], and insurance type is the main factor affecting affordability of these agents [20]. Our study showed that privately insured MM patients aged 65 years or older lived longer than patients did on Medicare. When considering confounding factors that may contribute to this difference we found that living in higher median income areas and/or receiving treatment in academic institutions were highly associated with improved survival, as also shown by others [16,21]. Furthermore, treatment that incorporated autologous HSCT led to similar survival, regardless of insurance or age. Non-transplanted patients, on the other hand, may be more dependent on continuous and often expensive medical treatment, in which affordability depends on the type of prescription plan coverage. MM treatment relies heavily on oral antineoplastic, prices of which have dramatically increased in the last 10 years [18]. There is a gap in the financial burden between patients with private vs. those with Medicare insurance [10], given that it is generally assumed that the former is associated with lower medication co-pays than the latter. Importantly, financial toxicity does not only affect quality of life but also medication adherence [19,22].

Our results are somewhat limited by lack of information on individual-based income and MM detailed baseline characteristics such as unfavorable cytogenetics, disease staging, and treatment history (i.e., doublet vs. triplet therapy). The information on the other staging systems such as Revised International Staging System (R-ISS) was not available. Furthermore, the staging information was not reported for a large portion of patients. However, given the large numbers of patients studied here, representing approximately 70% of all US patients with MM, it is unlikely that such variables would change our results.

Studies on the relationship between racial disparities and outcomes of MM patients have reported inconsistent results [20,23,24,25,26,27]. The influence of race on survival is complex and frequently superimposable with economic and geographic factors [16,28]. As indicated previously by Go et al. [21], a higher proportion of African-Americans had access to academic centers. One can speculate that the shorter distance from the treatment facility for blacks can mirror the geographical clustering of high black/white ratios in inner city areas where large cancer centers are frequently located. Due to the de-identified nature of NCDB data for cancer centers and patients, we could not examine this hypothesis. In our study, treatment that incorporated autologous HSCT led to similar survival, regardless of insurance, race, or age. That can be partially due to the fact that HSCT patients may be less dependent on continuous and often expensive medical treatment, in which affordability depends on the type of prescription plan coverage.

Accordingly, a surveillance, epidemiology, and end-results-based study of 10,161 MM patients younger than 65 years diagnosed between 2007 and 2012 showed that marital status, insurance type, and county median income were significantly associated with survival. Insurance status, however, was described as Medicaid, insured, or uninsured, likely reflecting the low number of Medicare patients in the study population. Interestingly, uninsured and Medicaid patients achieved similar overall survival [16]. This finding, also observed here, may be explained by the fact that uninsured patients at diagnosis may have subsequently applied and obtained Medicaid coverage.

Medical insurance reduces the financial burden of treatments, but there is significant variation and disparity between patients on private insurance and those on Medicare. State-enacted oral chemotherapy parity laws force private insurances to cover oral anticancer medications similar to office-administered treatments [29]. However these laws do not apply to patients on Medicare who are left with cost-sharing of an estimated 25% for these expensive drugs. Even after coming out of the coverage gap (also known as the “donut hole”) and entering catastrophic coverage where the cost-sharing becomes 5%, the out-of-pocket cost remains substantial, taking into account that the price of one medication may approach USD 200,000 per year [30]. In 2011 the mean out-of-pocket per patient per month cost for cancer patients (not myeloma-specific) enrolled in Medicare Part D who took oral anticancer medications was significantly higher (USD 832) than that of privately insured patients (USD 198) [18]. A study looking at the out-of-pocket costs over one year in 1900 MM patients showed that Medicare patients paid significantly more for oral thalidomide, lenalidomide, and intravenous bortezomib (USD 8824 vs. USD 12,568 vs. USD, 395, respectively) than patients overall (USD 4443 vs. USD 4766 vs. USD 3504, respectively) [31].

We compared outcomes of patients with private insurance vs. those with Medicare, and of those who received care in tertiary centers vs. in community centers. Data were not available specifically for patients treated at Veterans’ Administration facilities. Our study did not show any significant difference in survival of MM patients based on race.

Although the association between drug affordability and outcomes was not formally established or studied here, we speculate that the influence of median income on survival is a powerful surrogate. More efforts are needed to improve patients’ access to MM therapies and to improve outcomes of all patients, regardless of their economic situations. Insurance coverage is one area that needs improvement, especially in view of the high pricing of oral MM drugs. Patients need to be educated on the role of autologous HSCT. Furthermore, MM is a complex chronic disease and adherence to continuous treatment and appropriate follow-up are crucial. A multidisciplinary patient-based individualized approach is critical. This is more feasible in tertiary centers, which is likely one of the reasons for better clinical outcomes in higher volume centers.

## 4. Methods

### 4.1. Data Source

NCDB is a hospital-based registry sponsored by the American College of Surgeons and the American Cancer Society. It houses data on more than 34 million cancer cases collected from more than 1500 Commission on Cancer-accredited facilities representing 70% of cancer patients in the USA. Our analysis included all patients (*n* = 117,926) with MM (ICD-O 9732) diagnosed between 2005 and 2014. Patients with plasmacytoma (ICD-O-9731/9734) were excluded.

### 4.2. Study Variables

Insurance status identified patient’s primary insurance at time of diagnosis (changes in insurance were not available). Insurance status was not available for 2827 patients (2.4%). Patients of races other than white or black were grouped as “others” due to their low number. Individual income was not available. We used instead the median household income in the patient’s zip code area derived from year 2000 US Census data. Household income was categorized in four groups based on equally proportioned ranges among all US zip codes [32]. Education level was categorized in four groups according to the proportion of adults who did not graduate from high school in the patient’s zip code area based on year 2000 US Census (Level 1: >29%, level 2: 20–28.9%, level 3: 14–19.9%, level 4: <14%) [32]. The area of residence was categorized as metro, urban, or rural according to the United States Department of Agriculture (USDA) Economic Research Service [33]. Treatment facility type was categorized as comprehensive community cancer program (500 or more new cancer cases per year), community cancer program (100–500 new cancer cases per year), integrated network cancer program (part of a network that offers comprehensive services), and academic comprehensive cancer program (more than 500 new cancer cases per year) [32]. Facility volume was calculated by using the total number of MM cases reported by a facility to the database over the study period and dividing it by the total number of the study period years. Facility volumes were grouped as ≤10, 10–50 or ≥50 patient/year categories. Secondary MM identified patients were those who had one or more cancer prior to their MM diagnosis. Other variables included gender, Charlson–Deyo score [34], Durie–Salmon stage [35], transplant status, facility location, and distance traveled from patient’s residence to the treating hospital in miles.

### 4.3. Statistical Methods

The comparison of patient characteristics among insurance types was done using ANOVA for continuous measurements and Chi-square test for categorical factors. OS was measured from the date of diagnosis to the date of death, censored at the date of last follow-up for survivors. Survivor distribution was estimated using Kaplan–Meier methods, and difference in OS between groups was examined by log-rank test. The effect of continuous measurements including age, distance to medical facility, and facility volume on OS was estimated using Cox proportional hazard model. The effect of insurance type on OS was estimated using multivariable Cox proportional hazard regression after adjusting for the influence of confounding factors. Proportional hazard assumption for Cox model was examined using Schoenfeld residuals [36]. Statistical analysis was done using SAS version 14.1 (SAS Institute, Cary, NC, USA). All tests were two-sided and *p*-value ≤ 0.05 was considered statistically significant.

## 5. Conclusions

Here, we showed that economic factors and treatment facility type are major determinants of MM patient survival in the US.

## Figures and Tables

**Table 1:** Patient Characteristics.

Characteristic	Total Number of Patients	Medicare N (%), Median [Range]	Private Insurance N (%), Median [Range]	Medicaid N (%), Median [Range]	Not insured N (%), Median [Range]	Other gov. ins. N (%), Median [Range]
Total Number	115,099	62,340 (53)	41,340 (35)	6410 (5.4)	3796 (3.2)	1213 (1)
Age at diagnosis	67 (19–90)	74 (23–90)	57 (21–90)	58 (19–90)	57 (21–90)	61 (27–90)
Gender						
Male	62,798 (55)	33,071 (53)	23,438 (57)	3338 (52)	2155 (57)	796 (66)
Female	52,301(45)	29,269 (47)	17,902 (43)	3072 (48)	1641 (43)	417 (34)
Race						
White	87,263 (77)	49,313 (79)	31,472 (76)	3446 (54)	2214 (58)	818 (67)
Black	22,976 (20)	10,982 (18)	7910 (19)	2432 (38)	1347 (35)	305 (25)
Other	3616 (3)	1489 (2)	1416 (3)	449 (7)	193 (5)	69 (6)
Median income (zip-code-based) *						
<30 k	16,419 (15)	9178 (15)	4497 (11)	1712 (27)	858 (23)	174 (14)
30–34.9 k	19,878 (18)	11,258 (18)	6341 (15)	1258 (20)	784 (21)	237 (20)
35–45.9 k	30,160 (27)	16,717 (27)	10,394 (25)	1687 (26)	951 (25)	411 (34)
≥46 k	44,367 (40)	23,000 (37)	18,419 (45)	1567 (24)	1044 (28)	337 (28)
Education level (zip-code-based) ^						
Level 1	20,276 (18)	10,731 (17)	6021 (15)	2156 (34)	1175 (31)	193 (16)
Level 2	25,513 (23)	14,000 (22)	8546 (21)	1700 (27)	997 (26)	270 (22)
Level 3	25,223 (23)	14,011 (22)	9054 (22)	1215 (19)	623 (16)	320 (26)
Level 4	39,807 (36)	21,410 (34)	16,026 (39)	1153 (18)	842 (22)	376 (31)
Area of residence						
Metro	92,236 (83)	48,638 (78)	34,110 (83)	5437 (85)	3147 (83)	904 (75)
Urban	16,795 (15)	10,172 (16)	5159 (12)	739 (12)	485 (13)	240 (20)
Rural	2357 (2)	1502 (2)	689 (2)	83 (1)	55 (1)	28 (2)
Facility location						
Northeast	24,688 (22)	13,135 (21)	9466 (23)	1560 (24)	459 (12)	68 (6)
Midwest	29,936 (26)	17,379 (28)	10,342 (25)	1331 (21)	678 (18)	206 (17)
West	18,113 (16)	9032 (14)	7071 (17)	1252 (20)	482 (13)	276 (23)
South	40,834 (36)	22,722 (36)	13,479 (33)	1950 (30)	2036 (54)	647 (53)
Disease						
Primary	97,693 (65)	49,969 (80)	37,167 (90)	5929 (92)	3564 (94)	1064 (88)
Secondary	17,394 (15)	12,366 (20)	4167 (10)	481 (8)	231 (6)	149 (12)
Durie–Salmon Stage						
1	2118 (1.8)	954 (2)	1010 (2)	92 (1)	36 (1)	26 (2)
2	2970 (2.5)	1371 (2)	1326 (3)	152 (2)	85 (2)	36 (3)
3	7502 (6.4)	3297 (5)	3334 (8)	518 (8)	258 (7)	95 (8)
Not available	105,336 (89.3)	56,718 (91)	35,670 (87)	5648 (89)	3417 (90)	1056 (87)
Charlson/Deyo comorbidity score						
0	87,121 (76)	44,534 (71)	33,963 (82)	4683 (73)	2998 (79)	943 (78)
1	18,799 (16)	11,626 (19)	5347 (13)	1110 (17)	538 (14)	178 (15)
2	6405 (6)	4303 (7)	1477 (4)	403 (6)	158 (4)	64 (5)
3 or more	2774 (2)	1877 (3)	553 (1)	214 (3)	102 (3)	28 (2)
Hematopoietic stem cell transplant						
Yes	18,213 (16)	4651 (7)	11,942 (29)	1075 (17)	272 (7)	273 (23)
No	94,354 (84)	56,606 (91)	28,244 (68)	5175 (81)	3414 (90)	915 (75)
Facility type ^£^						
Community	10,460 (9)	6789 (11)	2713 (7)	573 (9)	270 (7)	115 (9)
Comprehensive community	45,428 (40)	28,007 (45)	13,962 (34)	1687 (26)	1292 (34)	480 (40)
Academic	45,990 (41)	20,791 (33)	19,834 (48)	3190 (50)	1677 (44)	498 (41)
Integrated network cancer program	11,693 (10)	6681 (11)	3849 (9)	643 (10)	416 (11)	104 (9)
Facility volume						
<10 patient/year	44,040 (38)	27,113 (43)	12,697 (31)	2271 (35)	1538 (41)	421 (35)
10–50 patient/year	55,648 (48)	29,354 (47)	20,390 (49)	3287 (51)	1978 (52)	639 (53)
>50 patient/year	15,411 (14)	5873 (9)	8253 (20)	852 (13)	280 (7)	153 (13)
Distance traveled						
Distance traveled (miles)	10 (0–4961)	8 (0–4961)	12 (0–3367)	7 (0–2439)	9 (0–2424)	14 (0–3329)
Traveled > 120 miles	4711 (4)	1964 (3)	2384 (5.7)	184 (2.8)	97 (2.4)	82 (6.7)

* <46 k vs. >46 k; ^ Level 1: >29%, level 2: 20–28.9%, level 3: 14–19.9%, level 4: <14%; ^£^ was categorized as comprehensive community cancer program (500 or more new cancer cases per year), community cancer program (100–500 new cancer cases per year), integrated network cancer program (part of a network that offers comprehensive services), and academic comprehensive cancer program (more than 500 new cancer cases per year).

**Table 2:** Multivariate Cox regression analysis (all patients).

Factor	Hazard Ratio (95% CI)	*p*-Value
Age (per year increase)	1.044 (1.039, 1.049)	0.000
Male vs. Female	1.06 (0.99, 1.14)	0.098
White vs. Black	1.07 (0.98, 1.17)	0.124
Primary Myeloma vs. secondary Myeloma	0.95 (0.86, 1.04)	0.242
Charlson–Deyo score (0 vs. 3)	0.48 (0.39, 0.59)	0.000
Charlson–Deyo score (1 vs. 3)	0.7 (0.57, 0.87)	0.002
Charlson–Deyo score (2 vs. 3)	0.92 (0.72, 1.16)	0.472
Other hospital types vs. Academic	1.49 (1.39, 1.59)	0.000
Zipcode-based median income: <46 k vs. ≥46 k	1.16 (1.08, 1.25)	0.000
Medicare vs. private insurance	1.09 (0.99, 1.2)	0.073
Medicaid vs. private insurance	1.59 (1.36, 1.87)	0.000
Not insured vs. private insurance	1.62 (1.32, 1.99)	0.000
Other gov. insurance vs. private insurance	0.95 (0.66, 1.39)	0.802

**Table 3:** Multivariable Cox model: based on subjects with age ≥65 years only.

Factor	Hazard Ratio (95% CI)	*p*-Value
Age (per year increase)	1.06 (1.055, 1.068)	<0.001
Male vs. female	1.05 (0.96, 1.15)	0.263
White vs. Black	1.05 (0.93, 1.18)	0.436
Primary myeloma vs. secondary myeloma	0.96 (0.86, 1.06)	0.387
Charlson–Deyo score (0 vs. 3)	0.52 (0.41, 0.68)	<0.001
Charlson–Deyo score (1 vs. 3)	0.77 (0.59, 1)	0.054
Charlson–Deyo score (2 vs. 3)	0.9 (0.67, 1.2)	0.482
Zipcode-based median income: <46 k vs. ≥46 k	1.09 (1, 1.19)	0.064
Other hospital types vs. academic	1.39 (1.28, 1.52)	<0.001
Medicare vs. private	1.04 (0.9, 1.2)	0.568
Medicaid vs. private	1.23 (0.87, 1.75)	0.246
Not insured vs. private	1.95 (1.26, 3.03)	0.003
Other gov. insurance vs. private	1.1 (0.6, 2.01)	0.763

## Data Availability

The data presented in this study are available on request from the corresponding author.

## Supplementary Materials

The following are available online at https://www.mdpi.com/2072-6694/13/4/590/s1, Figure S1: Kaplan–Meier estimation of overall survival by race.

## Abbreviations

MM	Multiple Myeloma
HSCT	Hematopoietic Stem Cell Transplant
