# Supplementary material for: Socioeconomic Factors and Survival of Multiple Myeloma Patients
Source: Cancers (Basel). 2021 Feb 3;13(4):590. doi: 10.3390/cancers13040590 (PMC7913279; doi:10.3390/cancers13040590)
Supplement: Supplementary file 1 [file cancers-13-00590-s001.pdf]

cancers

Supplemental Material

Socioeconomic Factors and Survival of Multiple Myeloma Patients

Kamal Chamoun, Amin Firoozmand, Paolo Caimi, Pingfu Fu, Shufen Cao, Folashade Otegbeye, Leland Metheny, Seema Patel, Stanton L. Gerson, Kirsten Boughan, Marcos De Lima and Ehsan Malek

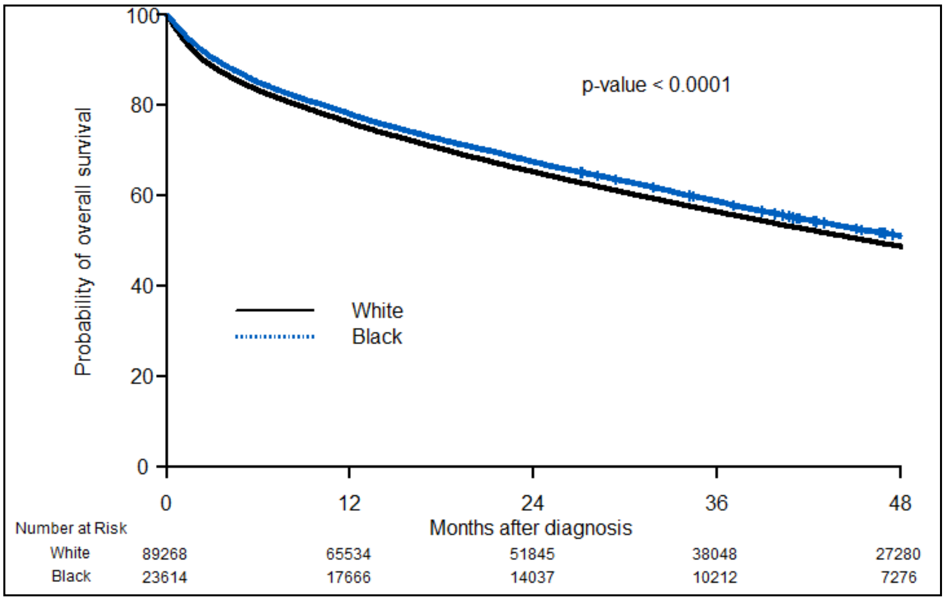

Figure S1. Kaplan–Meier estimation of overall survival by race.
